# Infection control in healthcare settings: perspectives for *mfDNA* analysis in monitoring sanitation procedures

**DOI:** 10.1186/s12879-016-1714-9

**Published:** 2016-08-09

**Authors:** Federica Valeriani, Carmela Protano, Gianluca Gianfranceschi, Paola Cozza, Vincenzo Campanella, Giorgio Liguori, Matteo Vitali, Maurizio Divizia, Vincenzo Romano Spica

**Affiliations:** 1 Unit of Public Health, University of Rome “Foro Italico”, Piazza Lauro De Bosis, 6, 00135 Rome, Italy; 2 Department of Public Health and Infectious Diseases, Sapienza University, Rome, Italy; 3 Department of Public Health and Cellular Biology, University “Tor Vergata”, Rome, Italy; 4 Hygiene and Epidemiology Chair, University “Parthenope”, Naples, Italy

**Keywords:** Healthcare-associated infections, Dental healthcare, mfDNA, Real time PCR, Sanitation procedures, Surveillance

## Abstract

**Background:**

Appropriate sanitation procedures and monitoring of their actual efficacy represent critical points for improving hygiene and reducing the risk of healthcare-associated infections. Presently, surveillance is based on traditional protocols and classical microbiology. Innovation in monitoring is required not only to enhance safety or speed up controls but also to prevent cross infections due to novel or uncultivable pathogens. In order to improve surveillance monitoring, we propose that biological fluid microflora (mf) on reprocessed devices is a potential indicator of sanitation failure, when tested by an mfDNA-based approach. The survey focused on oral microflora traces in dental care settings.

**Methods:**

*Experimental* tests (*n* = 48) and an “*in field”* trial (*n* = 83) were performed on dental instruments. Conventional microbiology and amplification of bacterial genes by multiple real-time PCR were applied to detect traces of salivary microflora. Six different sanitation protocols were considered. A monitoring protocol was developed and performance of the mfDNA assay was evaluated by sensitivity and specificity.

**Results:**

Contaminated samples resulted positive for saliva traces by the proposed approach (C_T_ < 35). In accordance with guidelines, only fully sanitized samples were considered negative (100 %). Culture-based tests confirmed disinfectant efficacy, but failed in detecting incomplete sanitation. The method provided sensitivity and specificity over 95 %.

**Conclusions:**

The principle of detecting biological fluids by mfDNA analysis seems promising for monitoring the effectiveness of instrument reprocessing. The molecular approach is simple, fast and can provide a valid support for surveillance in dental care or other hospital settings.

## Background

Healthcare-associated infections (HAIs) in medicine and dentistry are an issue of great concern for public health, as they represent the most frequent adverse effect during care delivery [1]. The global burden of HAIs remains unknown due to the lack of surveillance systems in several countries and to the absence of harmonized criteria for their diagnosis. However, on the basis of the available data, it can be estimated that each year hundreds of millions of patients are affected by HAIs worldwide, with an annual prevalence ranging from 3.5 to 12 % in high-income countries and at least 2–3 fold higher in low or middle-income countries [2].

Dental healthcare settings are associated with a risk of exposure to microorganisms both for dental workers and patients [3–5]. Microbiological hazards involve a wide number of microorganisms detected in saliva and gingival fluids as well as on contaminated dental instruments [6–10]. Considering unrecognized or under-reported cases it is assumed that the real threats of cross-transmission in dentistry are probably higher than that of other clinical settings [11]. Techniques for sanitizing reusable equipment are reported as key measures for controlling HAIs in dentistry [6, 12–18]. These techniques differ according to the reusable items: critical, semi-critical, non-critical items; in particular, critical and heat-tolerant semi-critical equipment should be sterilized by heat (autoclaving, dry heat, unsaturated chemical vapor), heat-sensitive semi-critical equipment should be processed by means of high-level disinfection, and non-critical items should be cleaned and/or disinfected using an hospital disinfectant registered by an official agency such as the United States Environmental Protection Agency (USEPA) [12]. All these procedures should be preceded by a decontamination treatment, applied to reduce residual biological risk for professionals who will perform subsequent treatments of sanitization [18, 19]. However, a lot of research performed in different countries showed that procedures are not harmonized [20]. Traditionally, the effectiveness of disinfection and sterilization protocols is checked by the use of conventional microbiology methods or by other means (i.e. engineering controls) according to the CDC guidelines on infection control [12, 18].

The development and diffusion of molecular techniques, e.g. Real Time PCR, conveyed several advantages in comparison to traditional culture-based methods, being less labor intensive and less time consuming; in addition, they can be tailored to be highly sensitive and specific, at reasonable costs [21]. Nucleic Acid Technologies, not only overcome the restrictions related to classical microbiological tests, but can also avoid the limitations posed by viable but non-culturable cells [22]. Thus, the potential application of molecular techniques represents a challenging opportunity to implement infection control, also in monitoring reprocessed devices exposed to biological fluids.

Recently, the identification and characterization of a biological fluid by the analysis of microflora DNA (*mf*DNA) has become a key technical approach in forensics [23]. A multiplex real-time PCR assay was developed based on the detection of the microflora genomic signature to identify different human body fluids as salivary, fecal and vaginal fluid [24].

Here, considering the hypothesis that oral *mf*DNA may be a suitable marker for residual salivary traces, we applied this analytical method to used and/or sanitized dental tools, with the final aim of testing an alternative approach to implementing surveillance.

## Methods

### Study design

**Fig. 1:**
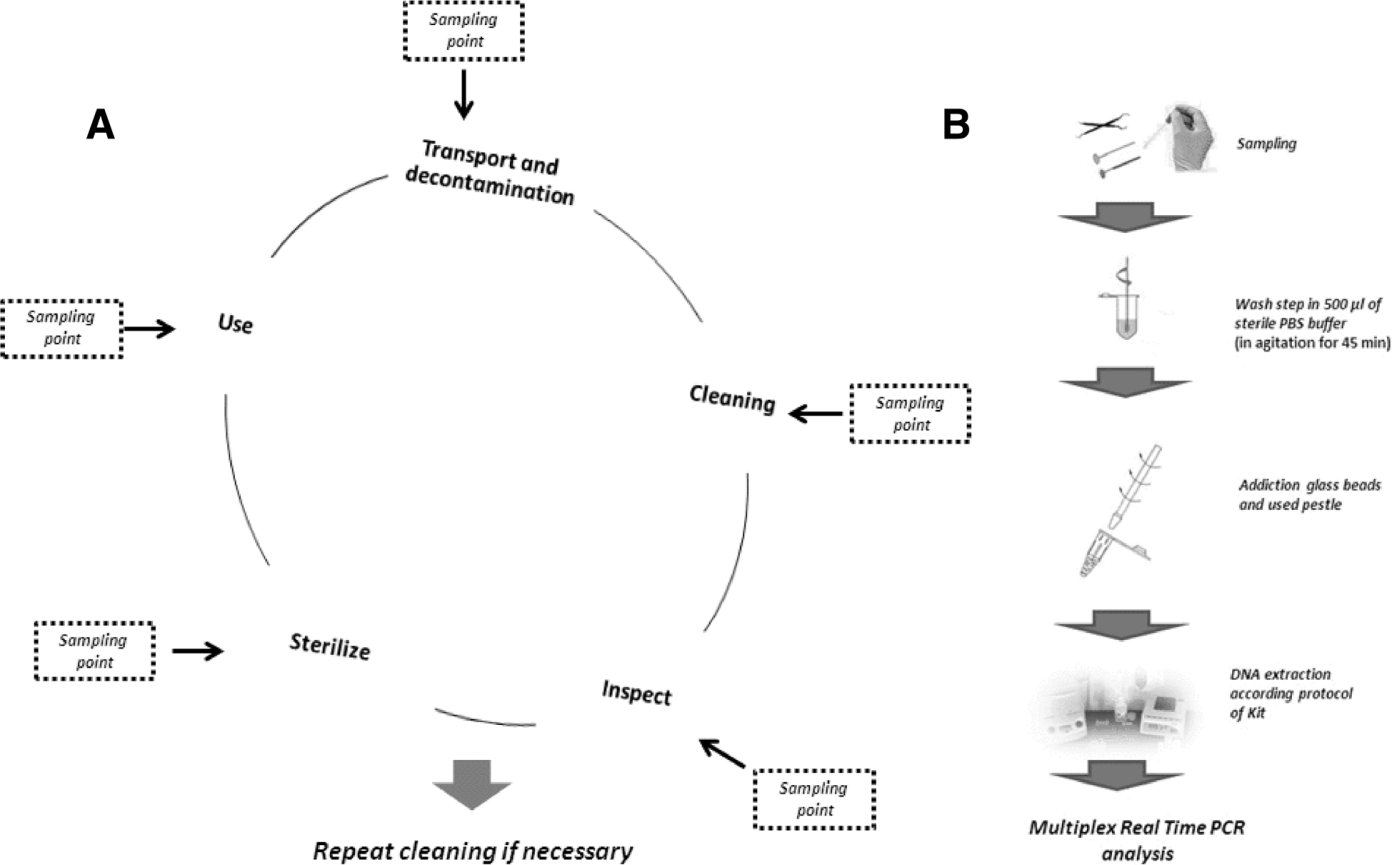
**a** Schematic representation of reprocessing procedures in dental care: critical steps (modified from [19, 20]); proposed sampling points are reported to monitor the different phases. Transport and decontamination are critical to assure both instrument and operator safety. They are performed by validated protocols and registered chemicals, following official guidelines or hospital approved protocols; cleaning by a washer-disinfector or manual steps is essential to remove those traces that could inhibit the sanitation efficacy. Inspection is visually performed by a magnifying device and is required to evaluate residual particulate contaminants, salt deposits or marked discolorations. Sterilization (autoclaving 121 °C for 30 min) is preceded by a packaging process. Dotted lines indicate sampling points for the monitoring of process main steps; **b** Sampling and analysis of *mf*DNA. In accordance with previously described protocols [23, 24], moistened sterile swabs were used to sample target surfaces. After washing in a PBS buffer, the bacterial wall was disrupted by glass beads using a mechanical pestle. DNA was purified by conventional kits and analyzed by real Time PCR

Two different strategies were applied, both considering stainless-steel dental mirrors as the standard reference: (I) In the *Experimental study*, tests were carried out on dental mirrors experimentally contaminated by two artificial salivary solutions; (II) In the “*In field” study*, tests were performed on dental mirrors actually in use in care settings. The sanitation procedures are summarized in Fig. 1 [18–20]. The sanitation treatment performed in the “*In field” study*, usually included a transient (3–12 h) storage step where used devices were collected in a bowl with disinfectant, before reprocessing. For this reason, we also sampled the walls and surfaces of these bowls.

### Experimental study

#### Artificial saliva composition

KCl 2.0⋅10^−2^ M, NaH_2_P0_4_ 1.4⋅10^−3^ M, NaHCO_3_ M, 1.5⋅10^−2^ M. Two different solutions (“White” and “Red”), were prepared to mimic oral fluids containing organic material, blood and bacteria and to test whether amplification was inhibited by hemoglobin, proteins or disinfectant residues, even after heating or drying steps: i) White solution: 50 % artificial saliva (pH 7.1), 45 % Tryptic Soy Broth, 5 % nuclease free water; ii) Red solution: 50 % artificial saliva (pH 7.1), 45 % Tryptic Soy Broth, 5 % defibrinated blood. *Streptococcus salivarius* cells were added to both solutions at a final concentration of 4x10^7^ cell/ml, mimicking real saliva conditions. 10 μl of the suspension (4x10^5^ cells) of Red solution or of White solution were spotted onto the surface of sterile dental mirrors and let completely dry. For each solution, 7 spots (6 processed samples and 1 unprocessed sample) were studied in triplicate. Moreover, two unprocessed samples spotted in triplicate with Red and White solutions (without bacteria) were also included as internal negative controls.

Six different sanitation protocols, selected in accordance with CDC protocols [12], and using chemical biocides authorized by the USEPA and the United States Food and Drug Administration (FDA) [20], were applied in triplicate on contaminated mirrors: (1) Full disinfection (without subsequent cleaning and sterilization): 5 min immersion without shaking in a solution containing 5 % Sporigerm (benzalkonium chloride 10 % w/w and orthophenylphenol 1 % w/w) in sterile demineralized water; (2) Partial disinfection, mimicking a shorter treatment: immersion for 5 s in a 0.5 % disinfectant solution; (3) Full disinfection: 10 min immersion without shaking in a solution containing 10 % Superacetic 10E (Peracetic acid generated from sodium percarbonate 42 % by an organic activator 25 %) in sterile demineralized water; (4) Partial disinfection, mimicking a shorter treatment: immersion for 5 s in a 1 % disinfectant solution; (5) Sterilization step in autoclave: (121 °C for 30’) without preliminary cleaning and disinfection; (6) Complete decontamination process: cleaning with detergents, 10 min disinfection with 10 % Superacetic 10E, followed by autoclave treatment at 121 °C for 30’.

Supplementary procedures were also considered (data not shown): i) disinfected/cleaned samples without subsequent sterilization; ii) positive and negative controls performed by applying Red or White solution containing and not-containing bacteria cells, without any subsequent sanitation treatment. Sampling was performed by a wipe test using sterile swabs (moistened with 80 μl of sterile water) rubbed over the surface of the dental mirrors, according to standard protocols [25]. Swabs were stored in dry conditions until processing.

Furthermore, in order to verify the efficacy of the sampling procedure and the possible loss of salivary material from swabs, we analyzed, in triplicate, swabs directly spotted with 10 μl of salivary solutions (Red and White), containing *Streptococcus salivarius* cells. In parallel, the same quantity of each solution was scraped directly onto two plates of Tryptone Soya Agar to test the presence of living bacteria and the number of Colony Forming Units (CFU).

### “In field” study

Eighty-three samples were collected from: dental mirrors after their actual use on patients (*n* = 64), disinfection bowl walls (*n* = 8), saliva from human volunteers (*n* = 11). Among the samples collected from mirrors used in patients, 22 were taken immediately after their use, before any sanitation; 22 after a subsequent step of preliminary disinfection in the temporary storage bowl (sampling was performed on the same dental mirrors by sampling different parts of the mirror e.g. front, rear); 20 after a subsequent step of complete sanitation (cleaning, disinfection, autoclave). Sterile swabs (*n* = 8) were rubbed over the bowl wall. All samples were collected in duplicate, anonymously and processed blindly.

Saliva human specimens were acquired from fully informed and autonomous volunteers accessing the clinical setting during operating hours. Samples (2–3 per session) were collected from patients presented on Wednesday and Thursday between h 10–12, for five following weeks, following procedures in accordance with the ethical standards of the responsible committee on human experimentation and the Helsinki Declaration. The study protocol was submitted to the Independent Ethics Committee and approved; Informed consent was required and no patient declined the participation to the research.

Each sample was analyzed with both molecular and microbiological approaches, scraping directly onto plates of Tryptone Soya Agar to test for the presence of living bacteria and the number of Colony Forming Units (CFU).

### DNA extraction and analysis of mfDNA by real time PCR

DNA extraction and amplification (Fig. 1) were performed as previously described [23]. Briefly, each DNA sample was evaluated in Real time PCR by means of three multiplex reactions: Mix Saliva (Mix_S), for identification of *Streptococcus salivarius*/*Streptococcus mutans*; Mix fecal traces (Mix_ES) for *Staphylococcus aureus*/*Enterococcus spp*.; Mix vaginal fluid (Mix_V) for *Lactobacillus crispatus*/*Lactobacillus gasseri*. Data C_T_ (cycle threshold) were analyzed considering clear amplification signals C_T_ < 35, weak 35 < C_T_ < 38, doubt signal for C_T_ > 38. For each sample, 10 μl of template DNA were amplified. In order to evaluate the sensitivity levels, 10-fold serial dilutions of *Streptococcus salivarius* DNA in both Red and White solutions were performed in triplicate and analyzed by Real-time PCR.

### Analysis of human DNA

The contamination with saliva also implies the presence of human DNA from cheek mucosa exfoliated cells. Indeed, in order to confirm the specific amplification of positive samples and/or the absence of mfDNA detection in negative samples, we also tested some contaminated, disinfected and sterilized mirrors for human DNA. Quantification was performed using the quantitative PCR assay Quantifiler Human DNA Quantification Kit following manufacturer instructions.

### Statistical elaboration

Quantitative data were summarized using the means and standard deviations of the three tests performed for each experiment. The performance of *mf*DNA analysis as a feasible tool for monitoring sanitation procedures was calculated in terms of sensitivity, specificity, false positive and false negative rates, efficiency and selectivity, as follows: Sensitivity: a/(a + b); Specificity: d/(c + d); False positive rate: c/(a + c); False negative rate: b/(b + d); Efficiency: (a + d)/n; Selectivity: Log_10_ [(a + c)/(a + b + c + d)] where “a” is the number of true positives, “b” is the number of false positives, “c” is the number of false negatives, “d” is the number of true negatives, “n” is the number of samples. False negatives were considered to be all those samples testing negative in used and/or not fully sanitized mirrors; false positive all those samples testing positive in unused and/or fully sanitized mirrors. Confirmation was done by verifying the presence/absence of: quantifiable extracted DNA, amplifiable human DNA, cultivable bacteria to confirm false and true negatives or positives, respectively: e.g. one sample was considered to be a false negative when the mirror was used, DNA was present, human DNA was detected, but saliva mfDNA tested negative.

## Results

The data from experimental tests performed on dental mirrors contaminated with artificial salivary solutions are summarized in Table 1. All contaminated or partially sanitized samples tested positive for the presence of saliva traces, (C_T_ < 35, range 20.2 to 29.5). Interestingly, only the samples which were completely processed tested negative. In a series of three replicate experiments, these data were consistent for both red and white solutions. Saliva traces were also identified in the cases of contaminated mirrors that were autoclaved, but not previously cleaned and disinfected. Conversely, fully disinfected and appropriately cleaned samples tested all negative for the presence of saliva traces, further supporting the role of this critical step (data not shown). All the internal negative controls without the addition of bacteria in the artificial saliva solutions were negative.

**Table 1:** Results from the *Experimental* trial: Real-time PCR analysis on samples, undergoing different sanitation treatment after experimental contamination by artificial salivary solutions, in presence (Red) and absence (White) of blood

		Treatment						
		No Treatment	Polyphenol 5 %		Superacetic 10 E		Autoclave at 121 °C 30 min	
	Contamination type		5 min	5 s diluited 1:10	10 min	5 s diluited 1:10	With cleaning and disinfection step	Without cleaning and disinfection step
Results time PCR (C_T_ mean ± SD^a^)	Red solution	20.2 ± 2.3	27.6 ± 5	26.6 ± 3	29.5 ± 2.3	26.7 ± 3	ND^a^	28.7 ± 1.2
	White solution	22.4 ± 0.2	28.8 ± 1.6	28.9 ± 2.4	25.9 ± 0.6	25.5 ± 0.5	ND^a^	28.5 ± 2.4

^a^ The data were expressed as the mean of threshold cycle (C_T_) of three independent replicates and corresponding standard deviation; *ND* Not Detected

**Fig. 2:**
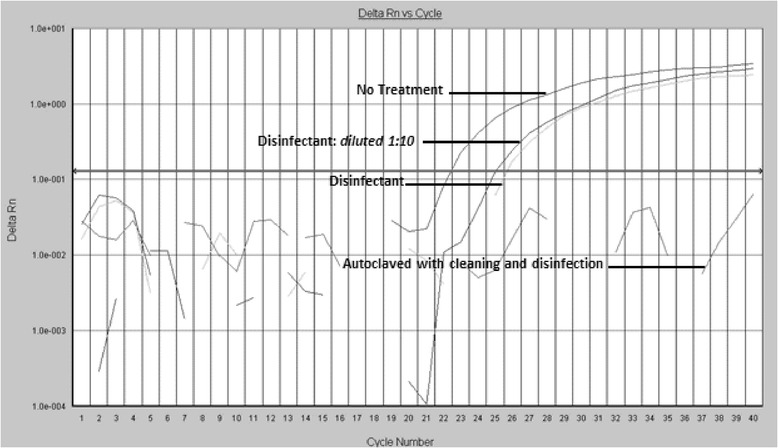
Real Time PCR amplification: exemplificative curve from *Experimental study* trial. Representative amplification plots. Analyses performed on used and disinfected dental mirrors. The comparison of the two plots shows a reduction of about 2–3 C_T_ cycle points after the immersion of the dental mirror in the disinfectant, but the complete absence of signal is observable only after the full reprocessing protocol, including cleaning, disinfection and autoclaving

In full or partial disinfection experiments, when using a lower concentration of disinfectant for a shorter treatment time, the comparison of the amplification curves showed a reduction of about two or three C_T_ cycle points in comparison to the untreated samples, corresponding to about one magnitude log in bacterial genomic units [26]. However, as shown in Fig. 2, the action of a complete or incomplete disinfectant treatment by itself never provided a negative result after amplification; by contrast, in the culture-based microbiological test, these samples always tested negative. This finding supports the efficacy of disinfection in inhibiting bacterial growth on culture plates (100 %), but also highlights the limitations of traditional microbiology in detecting incomplete sanitation or traces of residual bacteria, as shown by real time PCR.

**Fig. 3:**
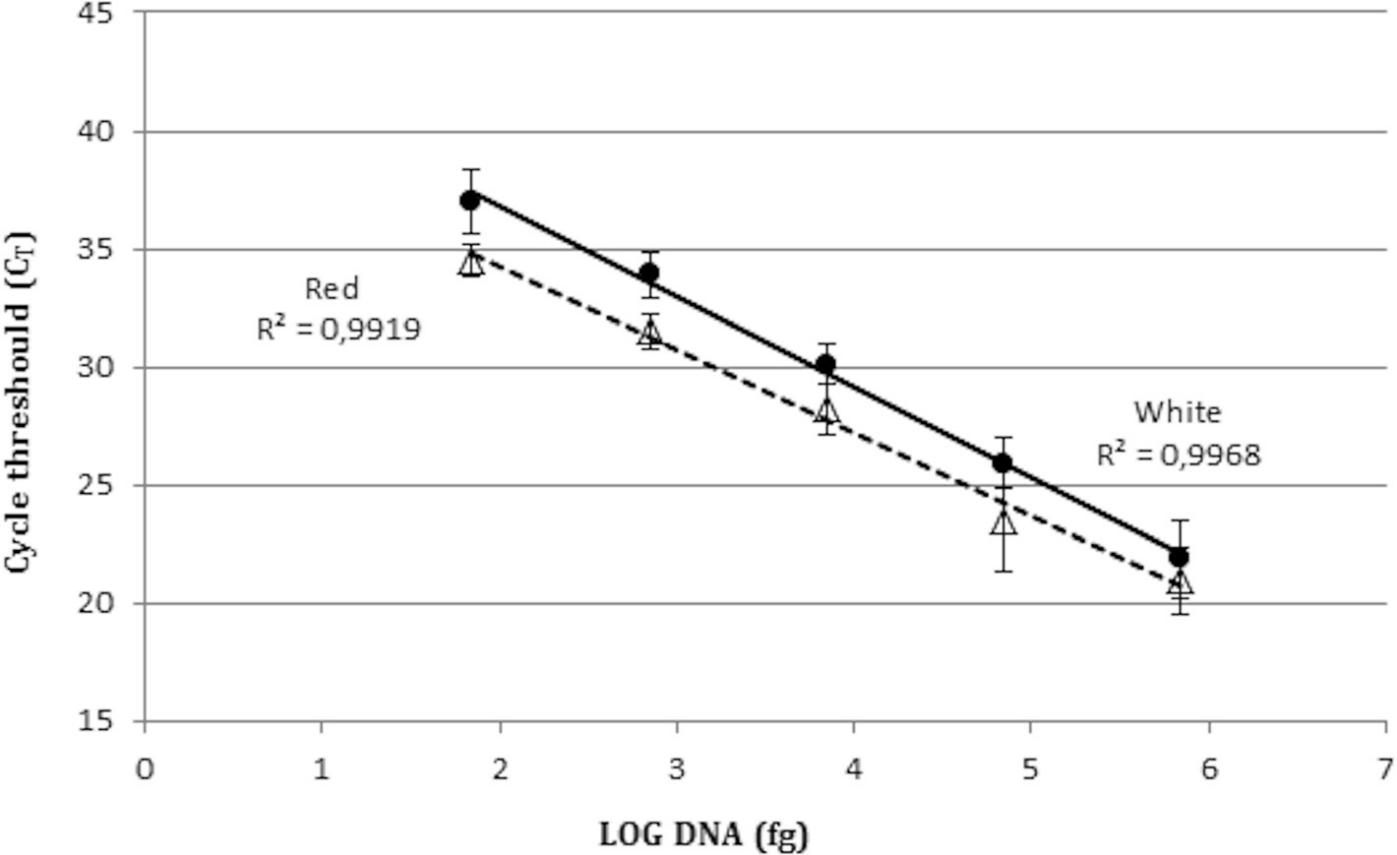
Sensitivity and linearity of the test. Real Time PCR of 10-fold serial dilutions of *S. salivarius* genomic DNA, extracted from Red and White solutions. Triangles: Red solution; Circles: White solution. Error bars represent standard deviation

Sensitivity and linearity limits of the proposed approach are shown in Fig. 3. No significant differences were observed in Red vs White solutions. The lower limit of detection was of 70 fg of template DNA corresponding to the genomic content of about 25–30 cells, in accordance with previous reports [27]. The set of data was characterized by elevated linearity and a correlation coefficient close to 1 (R_2_ = 0.99), both for Red and White solutions.

The results from “in field” analyses are reported in Table 2. All the samples correctly sterilized after complete reprocessing resulted clearly negative for Mix_S, as well as for all the other Real Time PCR Mix.

**Table 2:** Results from the *“in field”* trial: real time amplification mix for different biological fluids were applied on dental mirrors after use, after disinfection in bowl, and after the complete reprocessing procedure

Used (C_T_)					Bowl disinfected (C_T_)					Complete reprocessing (C_T_)				
*ID*	Mix_Es	Mix_S	Mix_V	Hu DNA	*ID*	Mix_Es	Mix_S	Mix_V	Hu DNA	*ID*	Mix_Es	Mix_S	Mix_V	Hu DNA
1u	-	**+**(36.9)	-	**+**(28.64)	1d	-	**+**(39.1)	-	**+**(27.26)	23	-	-	-	NA
2u	-	**+**(34.3)	-	**+**(27.27)	2d	-	**+**(34.8)	-	**+**(29.37)	24	-	-	-	NA
3u	-	**+**(37.8)	-	*NA*	3d	-	**+**(37.9)	-	*NA*	25	-	-	-	NA
4u	-	-	-	**+**(30.4)	4d	-	**+**(27.7)	-	*NA*	26	-	-	-	NA
5u	-	-	-	**+**(29.9)	5d	-	-	-	*NA*	27	-	-	-	NA
6u	-	**+**(36.2)	-	*NA*	6d	-	-	-	*NA*	28	-	-	-	NA
7u	-	-	-	**+**(26.8)	7d	-	-	-	*NA*	29	-	-	-	NA
8u	-	**++**(29.1;35.73)	-	**+**(26.26)	8d	-	**++**(29.1;35.7)	-	**+**(30.47)	30	-	-	-	-
9u	-	**++**(28;30.3)	-	**+**(26.7)	9d	-	**+**(36.1)	-	**+**(27.48)	31	-	-	-	-
10u	-	-	-	**+**(27.2)	10d	-	-	-	*NA*	32	-	-	-	-
11u	-	**+**(38.4)	-	**+**(31.33)	11d	-	**++**(31.3;33.1)	-	*NA*	33	-	-	-	-
12u	-	**++**(32.7;37.13)	-	*NA*	12d	-	-	-	*NA*	34	-	-	-	-
13u	**++**(30.12;23.21)	**+**(34.7)	-	*NA*	13d	-	-	-	*NA*	35	-	-	-	-
14u	**+**(32.58)	**++**(38.29;32.14)	-	**+**(28.89)	14d	-	**+**(34.3)	-	**+**(34.2)	36	-	-	-	NA
15u	**+**(35.72)	**+**(33.2)	-	*NA*	15d	-	**+**(34.5)	-	*NA*	37	-	-	-	NA
16u	-	**++**(35.7;32.5)	-	**+**(27.21)	16d	-	**+**(33)	-	**+**(29.19)	38	-	-	-	NA
17u	-	**+**(33.2)	-	*NA*	17d	-	**++**(35.7;36.8)	-	*NA*	39	-	-	-	NA
18u	-	**++**(35.78;36.64)	-	*NA*	18d	-	**+**(35.6)	-	*NA*	40	-	-	-	NA
19u	-	**+**(34.9)	-	**+**(27.21)	19d	**+**(25.2)	**+**(36.1)	-	**+**(27.21)	41	-	-	-	NA
20u	-	-	-	-	20d	-	-	-	*NA*	42	-	-	-	NA
21u	-	**+**(38.2)	-	*NA*	21d	-	-	-	*NA*					
22u	-	**+**(38.4)	-	*NA*	22d	-	-	-	*NA*					

(−) negative sample; (+) positive sample; (++) positive sample with two indicators of biological fluid (Mix_ES: *E. faecalis* and *S. aureus*; Mix_S: *S. salivarius* and *S. mutans*), C_T_ values are shown in brackets; NA = Not Applicable; u: used mirror; d: used mirror after immersion in the disinfectant of the temporary storage bowl; HuDNA: Human DNA; additional testing was performed only on borderline doubt samples selected with a C_T_ over 35

Out of the 22 “in field” used dental mirrors, the Mix_S was able to detect either *S. salivarius* and *S. mutans* in 77 %. Only three samples tested positive with the Mix_ES, and one of them was positive for *Enterococcus spp.* and *Staphylococcus aureus*. All the samples were negative at the Mix V.

More than half (60 %) of the dental mirrors immersed in the disinfectant bowl were still found to be positive for the Mix_S. However, the remaining 40 % of negative samples were already at a very low level of contamination after use (Ct >35) and only one case testing negative after use became positive (C_T_ = 27.7) after immersion in the disinfection bowl (sample 4d, see Table 2). In only three cases, the samples were positive for *Streptococcus mutans*, while one sample was positive at the Mix_ES, and with a double positivity both for *E. faecalis* and *S.aureus* Also in this set of the experiment, the samples were all negative for the Mix V.

The samples collected from the temporary storage bowl (*n* = 8) were all negative for Mix_ES and Mix_V; but one sample tested positive for both bacteria detectable by Mix_S, suggesting a possible sporadic contamination of the storage bowl.

91 % of the 11 swabs contaminated directly with salivary fluid from human volunteers tested positive with Mix_S, and negative with the other Mix.

As far as the human DNA tests are concerned, a selection (*n* = 20) of borderline samples was tested confirming that 19 contaminated and/or disinfected mirrors showed the presence of human DNA in the range 0.02–0.36 ng/μl (data not shown). Only one sample (20u) tested negative for both mfDNA and human DNA.

Consistently, all the sterilized mirrors were also negative for the presence of human DNA.

Based on the observed results, we calculated the sensitivity, specificity, false positive rate, false negative rate, efficiency and selectivity of the tests. As shown in Table 3, the results were respectively 81 %, 100 %, 0 %, 2, 82 % and −0.11 for “*in field*” samples when using the raw data (referring to the whole monitoring procedure: sampling-extraction-analysis), and 95 %, 100 %, 0 %, 0.5, 95 % and −0.05 after confirmation of false negative results (referring only to the analysis by a real time amplification method); while for dental mirrors experimentally contaminated with artificial salivary solutions, we obtained the same values for specificity, false positive rate, false negative rate, and selectivity, but observed a further increase in sensitivity (100 %) and efficiency (100 %). Taken together, these data support the effectiveness of the proposed approach.

**Table 3:** The performance of proposed approach was calculated in terms of sensitivity, specificity, efficiency and selectivity

Characteristics	Results	
	Experimental test	In field test^a^
Sensitivity	100 %	95 % (81)
Specificity	100 %	100 % (100)
Efficiency	100 %	95 % (82)
Selectivity	−0.06	−0.05 (−0.11)

^a^In brackets the values on raw data without confirming the true negatives samples

## Discussion

Increasing knowledge on cross-infection risks in dental healthcare [6, 12–18] has led to improved surveillance procedures in dental hygiene practice in the last years. Starting from the 1980s, CDC and other agencies have published and updated specific guidelines for reusable instruments, focusing on the cleaning/disinfection/sterilization flow and on the need for effectiveness and appropriateness, to obtain a “step by step” trustworthy sanitation protocol [12, 18–20]. Implementing the monitoring of sanitization procedures for reprocessing medical devices plays a fundamental role in assuring safety and preventing healthcare-associated infections.

We tested the application of a new molecular approach, based on the identification of residual traces of a biological fluid starting from the detection of its microflora components by mfDNA amplification. In comparison to traditional protocols based on bacteria indicators, this strategy requires an equipped molecular biology laboratory, but seems to carry several advantages. Firstly, it is independent of microbial culture requirements, allowing the detection of traces even after incomplete or unsuccessful reprocessing. Secondly, it is not based on pathogen identification, but on the search for microbial markers whose presence in biological fluids would indicate a possible occurrence of undesirable pathogens, including viruses or prions, thus suggesting a failure in the reprocessing chain. Moreover, the method based on bacterial DNA amplification has the advantage of starting from prokaryotic cells, that are in higher number in saliva in comparison to tissue exfoliated human cells and have a higher DNA resistance to environmental agents. Finally, the saliva microbial signature can be easily implemented based on microbiome achievements and technological advances, representing a promising approach to monitoring sanitation procedures. In comparison to traditional culture based methods, the main limits of this strategy include the availability of a molecular biology laboratory equipped with a real time PCR apparatus, with related costs; moreover, it is not possible to discriminate between live and dead cells; finally, not all the different steps can be evaluated, such as autoclaving performance or partial disinfection. However, it is important to consider that sterilization controls and disinfectant evaluation are already very well established following mandatory rules [18–20].

We searched for residual salivary traces by mfDNA analysis, in order to evaluate the extent of sanitation of reprocessed dental devices, collected after different treatments. For this purpose, we performed two kinds of evaluation: the *Experimental* test, based on trials carried out on dental mirrors, experimentally contaminated by two different artificial salivary solutions; and the “*In field*” test, performed on dental mirrors actually in use in everyday dental care settings. The main result concerns the demonstration of the feasibility of *mf*DNA analysis as a tool for monitoring sanitation efficacy in reprocessing dental instruments.

In the *Experimental* test, all the contaminated or partially sanitized samples tested positive for the presence of saliva traces, and only the completely processed samples were confirmed as negative. This result suggests that saliva traces, detected by amplification of *mf*DNA, can really represent a useful marker for monitoring sanitation. Moreover, the contamination was also identified in samples that were autoclaved without the mandatory cleaning and disinfection steps, further confirming its applicability in surveillance.

Full disinfection and appropriate cleaning represent the fundamental steps for saliva removal, following guidelines [18–20]. The physical or chemical sterilization step is essential to avoid further contaminations related or not to biological fluids, as well as to safely package and store the reprocessed medical tools [19].

The bacterial genome has a high environmental resistance to chemical and physical stressful conditions. This resistance could affect the differences between full and shorter treatments both for Red and White solutions (Table 1). Data reported in Table 1 show some variability in experiments conducted with Red solution, compared to those performed with White solution, however these differences were not statistically significant.

Interestingly, even after disinfection or autoclave treatment, both white and red solutions were detectable, showing that no amplification inhibition of artificial saliva was induced by hemoglobin or proteins or disinfectant residues, neither after heating or drying steps.

However, it should be emphasized that experimental results were obtained under a controlled situation, without interferences, such as the presence of other microorganisms, environmental agents and using an established load of *S. salivarius*. For this reason, we also applied the “*in field*” strategy considering different situations in a blind sampling collection. The proposed approach was successful also when used for “*in field*” assays, confirming the sanitation of the mirrors correctly reprocessed, and conversely showing the presence of salivary fluid on samples used and not treated, or used but partially or inappropriately processed.

In the “*in field”* test, we reported a slight reduction in sensitivity and efficiency, mainly due to false negatives resulting from used and not sanitized mirrors. In order to confirm this data we verified samples for the presence of amplifiable DNA, including human saliva traces and cultivable bacteria, excluding inhibitory effects or laboratory cross contaminations. Finally, only one sample out of a total of five proved to be a true false negative; since the mirror was used, DNA was present, human DNA was revealed, but saliva mfDNA was never detected. Another sample, indeed, had no DNA, probably due to a failure in sampling or in the extraction phase or because the mirror was not in contact enough and polluted with saliva. The other three samples showed a relevant bias in the microflora structure since they were collected from patients affected by Candidiasis, suggesting that *Candida* colonization (or the drug treatment) may have interfered with the levels of *S. salivarius* in saliva. Even if commensal existence of oral *Candida* species is not a rare condition [28], its concentration was usually much lower (three folds) than those recovered in these false negative samples. The possibility that a pathology or antibiotics may interfere with this mfDNA-based approach was already considered in forensics applications [29]. This limitation might be easily overcome by increasing the number of samples when monitoring a critical surveillance process. Moreover, the extension of the panel of oral bacteria markers, can further overcome this limit, enhancing the already high level of sensitivity.

The effectiveness of the general principle based on tracing saliva by mfDNA analysis was highlighted by the consistent negativity shown when using another PCR Mix addressed to the identification of other biological fluids (e.g. vaginal and colonic). The sporadic (4 out of 83) observed low positivity for *S. aureus* or *Enterococcus spp.* in saliva can be due to accidental contamination, rare but possible [30].

In everyday hospital practice, common disinfection bowls can temporarily join different medical devices, just after their use. When the method was applied to dental mirrors immersed in this container, a possibility of cross contamination emerged, as confirmed also by swab samples collected from the bowl liquid or surfaces, showing positivity for bacteria detectable by Mix_S. Saliva residuals were recovered both on dental mirrors disinfected or sterilized without preliminary cleaning and disinfection. The latter remarks are in accordance with several reports and further highlight the need for following all the required sanitation steps, as recommended in the specific guidelines [6, 14, 18–20]. This is a critical point that can also be overcome by the mfDNA monitoring [5]. Additional questions may arise for new pathogens or prion proteins that are undetectable by classical methods or could be more resistant to sanitation. This is especially relevant when the infected biological fluids are dried on the glass or metal surfaces of reusable tools, and/or would undergo only partial reprocessing [31]. Traditional methods based on microbial culture or single pathogen detection cannot be applied to monitor most of these events. Molecular tracing of biological fluids may overcome some of these limits related to viable but not cultivable species or due to the detection of microorganisms after disinfection treatments.

## Limitations of the method

The lack of a highly sensitive and specific gold standard to measure ‘perfect sanitation’ represented a main limitation to compare the effectiveness of the proposed method. Despite the lack of an optimal reference paradigm, we approached validation using classical microbiology, as traditionally performed in routine surveillance. Moreover, since risks related to incomplete sanitation are healthcare-associated infections, the detection of growing bacteria always is a fundamental test for hospitals practice and guidelines [12, 18, 20].

Other limitations of the proposed method include the availability of an equipped molecular biology laboratory and trained personnel. If reagents and consumables for real time PCR are readily available, to set up of a new laboratory would require relevant efforts and costs. However, most hospitals already have available real time pcr instruments for routine diagnostics. Finally, a major limitation of the method is related to the presence of dismicrobisms. Antibiotics, inflammatory diseases, oral disinfectants or even acute infections (e.g. Candidosis) may dramatically modify the microflora biodiversity, resulting in possible false negatives. However, considering the high sensitivity (>80–95 %), sampling of reusable devices on a large scale may overcome this limitations and support surveillance programs.

## Conclusion

The general principle of detecting residual saliva by microflora DNA amplification further shows the multifaceted complexity of monitoring the reprocessing process. The proposed approach based on tracing biological fluids by mfDNA seems to be a promising model and a feasible solution for infection control and prevention in dental healthcare or other hospital settings.

## Abbreviations

CDC, centers for disease control and prevention; CFU, colony forming units; C_T_, cycle threshold; FDA, food and drug administration; HAIs, healthcare-associated infections; mfDNA, microflora DNA; Real-time PCR, real-time polymerase chain reaction
